# Supplementary material for: False discovery rate estimation and heterobifunctional cross-linkers
Source: PLoS One. 2018 May 10;13(5):e0196672. doi: 10.1371/journal.pone.0196672 (PMC5944926; doi:10.1371/journal.pone.0196672)

# **Supporting Information**

## **S1 Derivation of formula**

The aim is to estimate how many false positives we have among the target-target matches. Known false positives in the form of target-decoy and decoy-decoy matches are used to model the score distribution of the unknown false target-target matches.

The equations use the following definitions:

X_DB_: for size of the initial search database

X_a_: entries in the database that can link to side 1 of the cross-linker

X_b_: entries that can link to side 2 of the cross-linker

X_ab_: entries that can link to both sites of the cross-linker

T: target entries

D: decoy entries

TT: target-target matches - i.e both sites of the link are from the target database

DD: decoy-decoy matches - i.e both sites of the link are from the decoy database

TD: target-decoy matches - i.e one site is from the target database one from the decoy database independent of order

ff(X) number of matches among X where both sides of the cross-link are false positive identifications

tf(X) number of matches among X where one sides of the cross-link is a false positive identifications and one is a true positive identification

Generalized FDR can be defined as:

1)
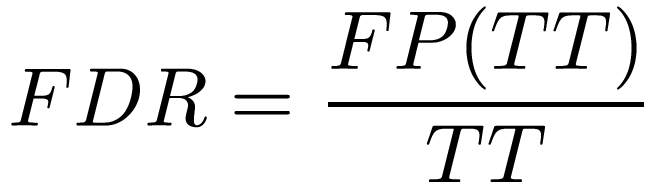


Where the false positives (
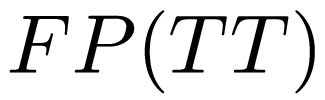
) are consisting of two distinct parts:

2)
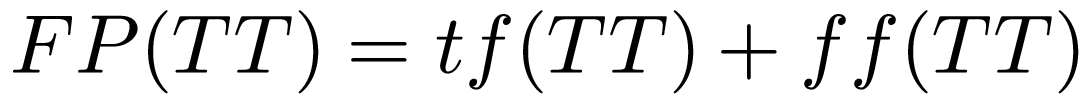


The matches with one side of the cross-link incorrectly identified (
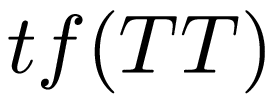
) and matches with two sides incorrectly identified (
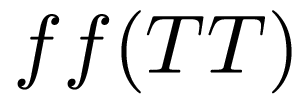
)

As the chance for a pair of matches to hit the target-target space randomly, is the same as falling into the decoy-decoy space (see Figure 1), we can estimate
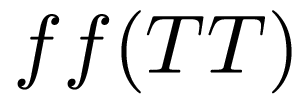
 as

3)
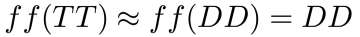


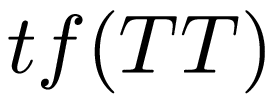
 needs to be estimated based on target decoy matches (*TD*)

4)
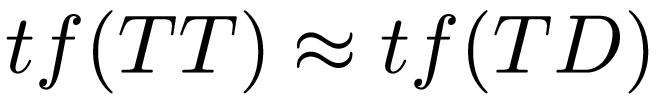


But TD also contains matches with two false identifications. So we need to reduce TD by these:

5)
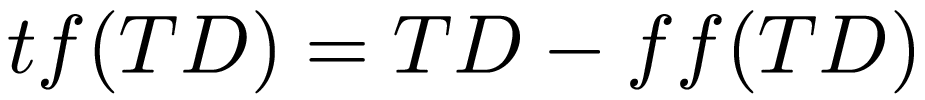


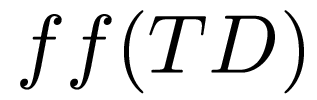
 again can be estimated by DD:

6)
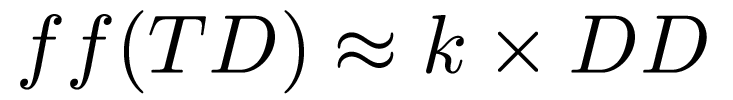


Where k is dependent on the size of the initial database for TD and DD:

7)
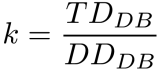


The size of
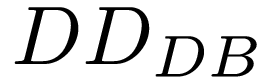
 is defined by

8)
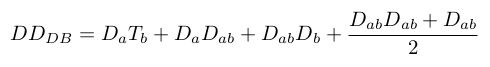


And
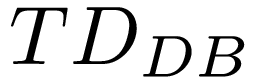
 by

9)
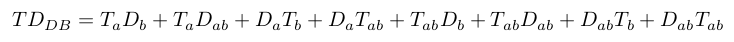


This results in k being

10)
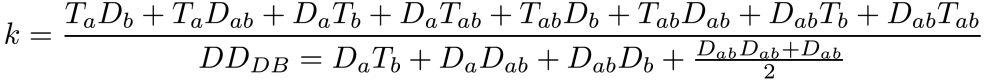


As we assume the target and decoy databases to be of equal size this can be changed to

11)
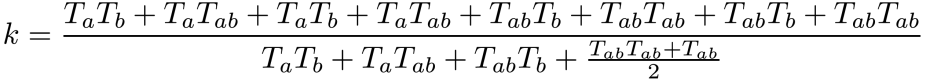


And reduced to

12)
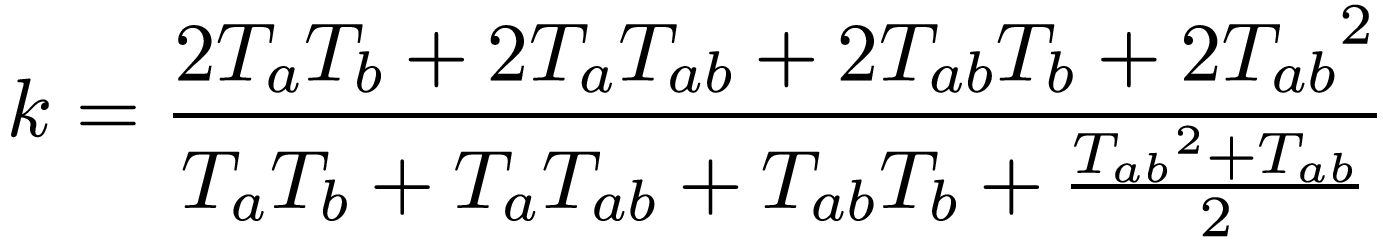


With


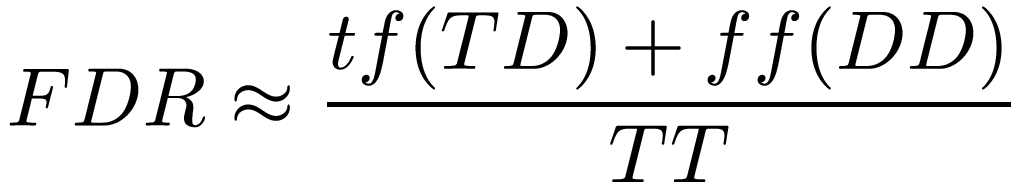


We can substitute the terms and get

13)
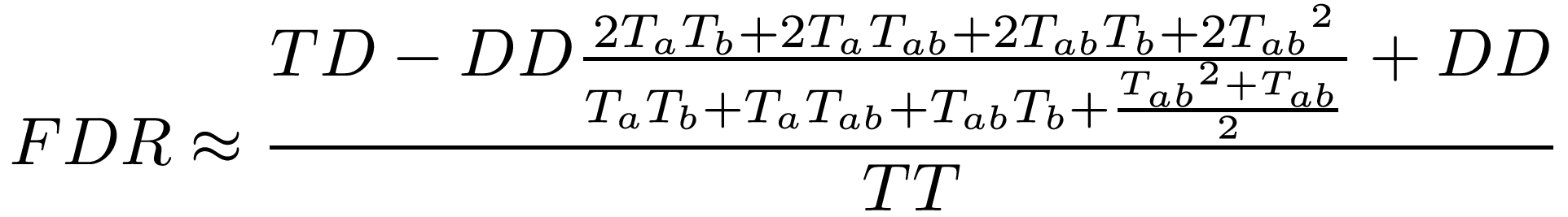


And somewhat reduce this to

14)
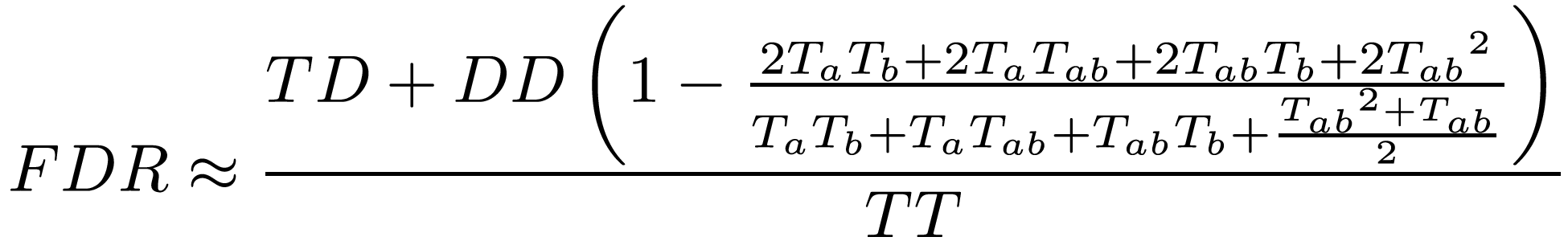

Supplement: S1 File — (DOCX) [file pone.0196672.s001.docx]
